# Regulation of Cancer Cell Responsiveness to Ionizing Radiation Treatment by Cyclic AMP Response Element Binding Nuclear Transcription Factor

**DOI:** 10.3389/fonc.2017.00076

**Published:** 2017-05-05

**Authors:** Francesca D’Auria, Lucia Centurione, Maria Antonietta Centurione, Antonio Angelini, Roberta Di Pietro

**Affiliations:** ^1^Department of Cardiac and Vascular Surgery, Campus Bio-Medico University of Rome, Rome, Italy; ^2^Department of Medicine and Ageing Sciences, G. d’Annunzio University, Chieti, Italy; ^3^Institute of Molecular Genetics, National Research Council-Pavia, Chieti, Italy; ^4^Ageing Research Center, CeSI, G. d’Annunzio University Foundation, Chieti, Italy

**Keywords:** cyclic AMP response element binding/activating transcription factor, radiotherapy, ionizing radiation, radio-resistance, radio-sensitivity, DNA repair, nuclear transcription factors, cancer

## Abstract

Cyclic AMP response element binding (CREB) protein is a member of the CREB/activating transcription factor (ATF) family of transcription factors that play an important role in the cell response to different environmental stimuli leading to proliferation, differentiation, apoptosis, and survival. A number of studies highlight the involvement of CREB in the resistance to ionizing radiation (IR) therapy, demonstrating a relationship between IR-induced CREB family members’ activation and cell survival. Consistent with these observations, we have recently demonstrated that CREB and ATF-1 are expressed in leukemia cell lines and that low-dose radiation treatment can trigger CREB activation, leading to survival of erythro-leukemia cells (K562). On the other hand, a number of evidences highlight a proapoptotic role of CREB following IR treatment of cancer cells. Since the development of multiple mechanisms of resistance is one key problem of most malignancies, including those of hematological origin, it is highly desirable to identify biological markers of responsiveness/unresponsiveness useful to follow-up the individual response and to adjust anticancer treatments. Taking into account all these considerations, this mini-review will be focused on the involvement of CREB/ATF family members in response to IR therapy, to deepen our knowledge of this topic, and to pave the way to translation into a therapeutic context.

## Introduction

Ionizing radiation (IR) induces loss of proliferative capacity as well as cell death by apoptosis and necrosis. Apoptosis can happen during interphase before division after the G2 block caused by IR (fast apoptosis) or after one or more divisions (late apoptosis). Some radiosensitive cells, such as lymphocytes, thymocytes, and intestinal crypt cells, undergo fast apoptosis without cell division after IR, while irradiated mouse leukemia cells undergo a G2 block and the apoptotic fraction begins to increase on the release from this G2 block (1–3). Furthermore, cells can respond by trying to counteract IR-induced damage to the membrane and to repair single- and double-stranded DNA breaks (respectively, SSB and DSB) by activating survival/signaling pathways. When the apoptosis ensues or the cell survives, genes that control complex pathways are activated (4). Amidst the proteins involved in cell responses to IR, a key role has been assigned to cyclic AMP response element binding (CREB) nuclear transcription factor (5, 6) and to members of the protein kinase C (PKC) family (7). CREB protein is a member of the CREB/ATF family of transcription factors that play a key role in the nuclear responses to a variety of external signals that lead to cell growth and proliferation, differentiation, apoptosis, and survival (8–10). Moreover, CREB has been also implicated in the adaptive response and, in particular, in the immune system balance (11–14). CREB protein is a 43-kDa basic leucine zipper (bzip) transcription factor composed of a C-terminal basic DNA binding domain, an adjacent leucine zipper domain, and a kinase-inducible transcriptional activation domain. The activation of CREB depends not only on the cell type but also on the stimulus, which has been administered (12, 15–18). CREB activation results from different posttranslational modifications, the main of which is the phosphorylation of serine-133 (Ser-133) that is mediated by several factors (19, 20). The signaling molecules responsible for CREB Ser-133 phosphorylation include Akt/protein kinase B (15, 21). Akt stimulates target gene expression *via* CREB in a phospho-Ser-133-dependent manner, promoting cell survival in response to growth factor stimulation (22). PKCs belong to a class of serine–threonine kinases, including at least 12 closely related iso-enzymes that have distinct, and in some cases opposing, roles in cell growth, differentiation, apoptosis, and survival (23). Among these 12 iso-enzymes, PKC delta is known for its activation in response to a variety of genotoxic stresses (24). PKC delta activity is controlled not only by binding diacylglycerol or phorbol ester but also by other molecular mechanisms, such as phosphorylation and proteolysis. In particular, an activation loop, a turn, and a hydrophobic motif site have been demonstrated at Thr505, Ser-643, and Ser-662, respectively, and these sites are phosphorylated *in vivo* (25). PKC can also undergo phosphorylation on tyrosine residues, such as Tyr-52, Tyr-155, Tyr-187, Tyr-311, Tyr-332, and Tyr-565, depending on the cell stimulus (26). Moreover, a catalytically active 40-kDa fragment of PKC delta arises from the proteolysis in cells exposed to IR, DNA damaging drugs, or anti-FAS antibody (27–31).

Based on these findings and on the complex and various molecular interactions involving CREB/ATF family members, this mini-review will be focused on the involvement of CREB/ATF family members in response to IR exposure to deepen our insight into this matter and contribute to the understanding of CREB-mediated radio-resistance/radio-sensitivity mechanisms. In fact, the knowledge of apoptotic and survival pathways recruited in tumor cells may be useful to establish targeted therapies by combining selective inhibitors or stimulators of fundamental signaling proteins with conventional chemotherapy, hormone therapy, and radiotherapy (RT) regimens.

## The Cellular Response to IR

Cancer cells respond to IR in a heterogeneous manner depending on their intrinsic properties (DNA repair capability, proliferation status) or extrinsic environment (degree of hypoxia within the tumor population) (32). In other cases, the degree of radio-sensitivity depends on the cell heterogeneity within the tumor mass. Both *in vitro* and *in vivo* clonogenic assays were used in early studies to address various determinants of tumor radio-response (33). These investigations lead to the identification of cells capable to initiate the growth of new tumors after *in vivo* transplantation. These cells were named tumor clonogens, cancer-initiating cells, or cancer stem cells, and their frequency inside a tumor mass was related to radio-resistance (32).

Ionizing radiation-induced damage leads to the activation of complex signaling cascades, inducing with different mechanisms DNA repair, stress response genes, cell-cycle arrest, cell death, or cell survival (34). A number of key molecules involved in different signaling pathways are modulated by IR, as recently detailed in a comprehensive review (35). The activation of these molecules ultimately results in altered expression of series of target genes, whose promoters or enhancers may contain binding sites for one or more transcription factors, able, in turn, to influence the transcription of multiple genes. Among these factors, NF-κB is widely recognized for its antiapoptotic and pro-survival effects possibly leading to radio-resistance (36). NF-κB has been demonstrated to be a downstream target of DNA DSB-activated [ATM kinase- and DNA-dependent protein kinase (DNA-PK)-dependent] pathway since it is activated following ATM-mediated phosphorylation and ubiquitin-dependent degradation of IκBα. DNA-PK activates NF-κB in a different manner since it is able to hyper-phosphorylate IκBβ that binds to NF-κB in the nucleus, thus preventing the binding of IκBα to NF-κB (37). In spite of these well-characterized mechanisms, it has been demonstrated that different IR doses are able to recruit different signaling pathways in different systems to cause activation of NF-κB (38). Interestingly, different levels of constitutive NF-κB activity in colorectal cancer (CRC) cells show a strong correlation with different levels of radio-sensitivity and spontaneous apoptosis (39). NF-κB together with c-jun and c-fos are considered as immediate response-genes transcription factors since they are switched on within minutes after IR, possibly thwarting the general downregulation of transcription upon irradiation and allowing privileged transcription of specific genes. NF-κB and p300/CBP (CREB-binding protein) have been involved in the rescue effects of irradiated cells (40). Both of them are downstream targets of PI3K/Akt pathway, and both of them are able to trigger cell death or cell survival. In a just-published paper of our research group, we highlighted the involvement of CREB/ATF family members in the radiation response of two different lymphoma cell lines with a different level of radio-sensitivity (Daudi and Ramos) suggesting a proapoptotic role of CREB and, surprisingly and unexpectedly, a pro-necrotic role of NF-κB after 3-Gy IR dose (6). Other authors have recently demonstrated that CREB is phosphorylated in response to IR through p38 MAPK pathway in a time- and dose-dependent manner. They also showed that the inhibition of CREB transactivation by decoy oligonucleotides significantly increases the radio-sensitivity of multiple human cancer cell lines (AGS, MCF-7, HeLa, SaOs-2, DU-145, and HepG2) (41). Furthermore, cancer cells that ectopically expressed dominant-negative mutant CREB (KCREB) or were treated with p38 MAPK inhibitors displayed a higher sensitivity to IR than wild-type parental cells or control-treated cells. In light of this evidence, CREB appears to protect tumor cells from IR and, thus, the combination of CREB inhibition plus IR could be evaluated as a possible RT approach in some cellular systems. Interestingly, CREB-dependent radio-resistance was also shown in normal cells like primary human fibroblasts that are capable to weather high doses of IR and to avoid IR-induced apoptosis. To identify possible antiapoptotic pathways in human fibroblasts, Bluwstein et al. (42) carried out a kinetic pathway analysis based on reverse phase protein arrays combined with extensive western blot analysis. Through these methods, these authors showed the activation of DNA damage–response networks (e.g., phosphorylation of MKK3/6, p38, MK2, p53, Chk1, and Hsp27), pro-survival molecules (CREB, MEK-ERK, PKC), and antiapoptotic markers (Bcl-2 and Bad). In particular, they observed that the inhibition or downregulation of PKC in primary human fibroblasts caused IR-dependent downregulation of the identified pro-survival pathways, including CREB, and antiapoptotic pathways, like Bad and Bcl-2, leading to proliferation arrest and apoptosis (19, 21) and suggesting a regulatory function of these pathways in response to IR. The radiation damage to biological systems is caused by the type of radiation, the total dose of radiation, the dose rate, and the region of the body exposed. IR-induced cytotoxicity in mammalian cells involves various modes of cell death, including apoptosis (type 1 cell death), necrosis (oncosis), autophagy (type 2 cell death), accelerated senescence, and mitotic catastrophe (43–45). The different types of cell death occur both *in vitro* and *in vivo* in response to IR in cancer cells as well as in normal cells (46). The cellular choice of a specific mode depends on various factors, including the specific cell type involved, the dose of IR absorbed, and whether the cell is proliferating and/or transformed (35). Increased expression of p53 (in its phosphorylated or acetylated form) is certainly a critical step in mediating the cellular response to IR-induced DNA damage (34). In fact, p53 activation leads to *de novo* synthesis of proapoptotic molecules that mediate intrinsic (e.g., Bax, Puma) or extrinsic (e.g., Fas) apoptotic cell death. Moreover, p53-dependent induction of p21/waf1 or the upregulation of other cell-cycle inhibitory proteins (e.g., p16 INK4a) is able to interfere with cell-cycle machinery resulting in accelerated senescence. The precise mechanisms for the biological selection of a specific pathway of cell death following IR exposure have not been established unequivocally. Additionally, the mechanisms through which IR causes death of the cells directly affected by IR differ from those through which bystander cells undergo cell death. Mitotic catastrophe is the main form of cell death induced by IR that results from premature or inappropriate entry of the cells into mitosis. It has been recently proposed that it could result from a combination of deficient cell-cycle checkpoints (in particular the DNA structure checkpoints and the spindle assembly checkpoint) and cellular damage (47). Cell death occurring during the metaphase/anaphase transition (mitotic catastrophe) is characterized by the activation of caspase-2 and/or mitochondrial membrane permeabilization (MMP) with the release of cell death effectors, such as apoptosis-inducing factor and the caspase-9 and caspase-3 activator cytochrome *c*. It is still debated whether mitotic catastrophe can be considered a special case of apoptosis or a completely different phenomenon. In favor of the first hypothesis, findings obtained by Castedo et al. (45) in HeLa cell lines forming syncitia demonstrated that cells died during the metaphase of the cell cycle as a result of the activation of caspase-2 upstream of MMP and cytochrome *c* release, leading to caspase-3 activation and chromatin condensation. By contrast, Roninson et al. (48) argued that mitotic catastrophe would be fundamentally different from apoptosis since the prevention of apoptosis by overexpressing, for example, Bcl-2 in etoposide-treated HeLa cells (49) or by overexpressing MDR in irradiated tumor cells (50) can actually enhance the frequency of catastrophic mitoses.

## IR and Hematological Malignancies

Myelodysplastic syndrome (MDS) is a group of blood disorders where hematopoietic stem cell undergoes an abnormal maturation and differentiation into one or more blood lineages. Disease progression is related to an increased genomic instability, and a high number of patients go on to develop acute myeloid leukemia (51–53). Primarily a disease of the elderly, it can also develop following chemotherapy. It has been reported that CBP heterozygous mice have an increased incidence of hematological malignancies, and it has been shown that CBP is one of the genes altered by chromosomal translocations found in patients suffering from therapy-related MDS (54). This evidence has led Zimmer et al. (55) to investigate whether hematopoietic tumor development in CBP+/− mice is preceded by a myelodysplastic phase and whether it could uncover molecular mechanisms that might contribute to its development. In particular, they observed that CBP+/− mice invariably develop myelodysplastic/myeloproliferative neoplasm within 9–12 months of age, are hypersensitive to IR, and show a marked decrease in PARP1 activity after IR. In addition, protein levels of XRCC1 and APEX1, key components of base-excision repair mechanism, appear reduced in CBP+/− cells not exposed to IR or upon targeted knockdown of CBP levels. This evidence provides validation of a novel myelodysplastic/myeloproliferative neoplasm mouse model and, more importantly, point at defective repair of DNA damage as a contributing factor to the pathogenesis of this currently incurable disease (55). In line with this evidence, the role of p38 MAPK in activating CREB metabolic pathway supported the events leading to erythroid differentiation (56). Furthermore, Cataldi et al. (57) demonstrated the effect of IR on PKC delta and Akt/CREB signaling pathways in Jurkat T human lymphoblastoid cells, known for being sensitive to IR (58, 59). More recently, our research group has shown the involvement of CREB family members in the IR response of Ramos B lymphoma cells, an Epstein–Barr virus-negative cell line derived from an American Burkitt’s lymphoma (6). In this case, CREB activation was unexpectedly linked to apoptotic cell death induced by IR whereas the early induction and activation of NF-κB1 in Ramos cells was associated with necrotic cell death and cell-cycle regulation (6). In another experimental model (represented by K562 erythro-leukemia cell lines), an IR dose of 1.5 Gy was considered as suboptimal, whereas a dose of 6 Gy was identified as the threshold dose above which cells lose clonogenicity, and the apoptosis rate increases (5). Interestingly, 6 Gy is the suboptimal dose delivered in RT protocols for the treatment of human hematological tumors, whereas it induces evident damage in many cancer cell lines (58). As previously demonstrated, K562 are radio-resistant human leukemia cells that express a high peroxiredoxins (Prx) stability (60). Prx are an extended family of small antioxidant proteins that conserve a thioredoxin-dependent catalytic function contributing to cell protection from reactive oxygen species, which are one of the deleterious intracellular effectors of IR damage. In fact, IR passing throughout living tissues generates reactive free radicals that can interact with critical macromolecules, such as DNA, proteins, or membrane lipids, inducing cell damage and, potentially, cell dysfunction and death (61).

## IR and Gastrointestinal Cancer

The gastrointestinal epithelium (GI) is very sensitive to IR and chemotherapy (62), and is prone to IR-induced malignancy (63). CREB has a recognized role in neo-oncogenesis occurring in T-cell and myeloid leukemia, hepatocellular carcinoma, melanoma, clear cell sarcoma, lung adenocarcinoma, as well as CRC (64). Various external stimuli can induce CREB activation *via* various kinase pathways, including stress networks. These signaling pathways lead to CREB phosphorylation at Ser-133, causing interaction with co-activators, including CBP or p300 (65, 66). Ultimately, p300 and CBP are not only CREB co-factors but are also able to activate additional transcription factors, including Myb. Myb is a fundamental transcription factor for proliferation and differentiation of the intestinal epithelium (67). The transcriptional coregulator p300 might integrate the co-activation and transcriptional activities of both phospho-CREB and Myb, enabling finely tuned cellular responses in the GI. Sampurno et al. (68) carried out a study in which an inducible CREB knockout (KO) model was used to define the role of CREB in the GI tract of adult mice under both homeostasis and stress conditions. Extensive phospho-CREB activation was shown in Apc (Min/+) mouse adenomas. Furthermore, in CRC, phospho-CREB was found in most cells associated with elevated CREB-target genes, including MRP2 (multidrug resistance-associated protein 2) involved in the vesicular transport of a platinum-based anticancer drug known as Oxaliplatin, used in the chemotherapy treatment of CRC. These observations lead the authors to hypothesize a role for CREB in GI malignancy but not in GI homeostasis where Myb appears to be critical (69). The same authors showed that defects in cell proliferation are sometimes revealed or exacerbated by stressing the GI epithelium with IR, as it was observed in Myb-mutant mice (69), although Myb expression itself was unaffected in CREB KO GI mice not exposed to IR. In another study performed in rat colon transversum, Pejchal et al. (70) demonstrated that during the first 24 h after IR the activated forms of ATF-2 and CREB participate at the cellular response after whole-body gamma IR within the low-dose range of 0.25–1 Gy and up to 10 Gy. Based on these findings, these authors proposed the detection of phospho-ATF-2 (Thr-69/71) and phospho-CREB (Ser-133) as a prospective bio-dosimetric tool for irradiated enterocytes *in vivo*.

## IR and Lung Cancer

Lung cancer is a malignant tumor arising from the respiratory epithelium that represents a leading cause of cancer death in both males and females worldwide. Lung cancer is divided into two morphologic groups: small cell lung cancer and non-small cell lung cancer (NSCLC). In spite of the number of therapeutic approaches (surgery, RT, and/or chemotherapy), the prognosis of lung cancer remains dismal, with a 5-year survival rate for NSCLC of around 15% (71). Thus, much research has been devoted to improving the efficiency of chemotherapy and RT based on a better understanding of the molecular mechanisms underlying cell proliferation and death (34, 72). In recent years, CBP and its homolog p300 have been demonstrated to play a role in respiratory epithelial tumorigenesis (73), and an increased expression in CREB protein has been associated with poor survival in NSCLC (74). However, controversial observations have been published regarding the role of CREB pathway in response to RT of lung cancer cells. In fact, Kim and Juhnn (72) demonstrated that cAMP signaling reduces sirtuin 6 (SIRT6) expression in human NSCLC by activating the PKA and CREB pathways. SIRT6 is able to remove acetyl groups from histones and to regulate genomic stability and cell viability, and when it is reduced, IR-induced apoptosis of NSCLC cells is increased. Moreover, Choi et al. (75) investigated the effect of the G-protein alpha signaling system on IR-induced apoptosis of p53-null H1299 lung adenocarcinoma cells, linking the increase in IR-induced apoptosis to the upregulation of Bak mediated by CREB and AP-1. By contrast, Cho et al. (76) reported that podophyllotoxin acetate (PA), a new anticancer drug candidate, inhibits IR-induced activation of CREB1/signal transducer and activator of transcription 3 (STAT3) pathway leading to the enhancement of apoptosis of wild-type p53 A459 NSCLC cancer cells, especially when used in combination with other chemotherapeutic compounds. In addition, they reported that IR increases migration/invasion and epithelial/mesenchymal transition (EMT) of A459 by increasing CREB1 and STAT3 activation and that PA blocks EGFR–p38/ERK–CREB-1/STAT3–EMT pathway to inhibit IR-induced invasion/migration. Taken together, these findings confirm that CREB/ATF family proteins behave as stress response signaling molecules but with pleiotropic effects depending on different experimental systems characteristics.

## IR and Prostate Cancer

Radiotherapy is a first choice therapy when prostate cancer is a localized mass. Although some patients are good responders to the treatment, nearly 70% of the patients experience recurrent tumors. However, the molecular mechanisms underlying tumor recurrence remain largely unknown. Deng et al. (77) showed that fractionated IR induces differentiation of LNCaP prostate cancer cells into neuroendocrine (NE)-like cells. These cells are involved in prostate cancer progression, androgen-independent growth, and poor prognosis. Further analyses revealed that CREB and ATF-2 function as transcriptional activator and repressor, respectively, of NE-like differentiation and that IR induces NE-like differentiation by increasing the nuclear content of phospho-CREB and the cytoplasmic accumulation of ATF-2. Consistent with these notions, stable expression of a non-phosphorylatable CREB or a constitutively nuclear-localized ATF-2 in LNCaP cells inhibits the IR-induced NE-like cell differentiation. In a recent paper, Suarez et al. (78) showed the increased activation of CREB protein during the course of fractionated IR-induced NE differentiation (NED). To determine whether targeting NED could be explored as an IR sensitization approach, these authors employed two CREB targeting strategies: (i) CREB knockdown and (ii) overexpression of ACREB, a dominant-negative mutant of CREB. Their results showed that ACREB expression increased fractioned IR-induced cell death and sensitized prostate cancer cells to IR. Consistent with this, knockdown of CREB also inhibited fractioned IR-induced NED. Interestingly, the authors observed that CREB targeting primarily increases IR-induced pre-mitotic apoptosis. Taken together, these results suggest that targeting CREB could be considered as an IR sensitization approach for prostate cancer treatment.

## Conclusion

Understanding the specific cellular mechanisms and signal transduction networks responsible for radio-resistance or radio-sensitivity is important in improving human cancer RT since it may help in establishing more efficient targeted therapies by combining selective inhibitors or stimulators of key signaling proteins with conventional RT. In this regard, different potential therapeutic targets have been identified in modulating IR sensitivity of various malignancies among which poly(ADP-ribose) polymerase (PARP), DNA-PK, DNA ligase IV, Mre11-Rad50-Nbs1 (MRN complex), and checkpoint kinase (79). Moreover, although this is outside the focus of this paper, a number of agents not directly involved in DNA damage repair or DNA damage response have been demonstrated to increase radio-sensitivity by inhibiting homology directed repair, many of these, including inhibitors of histone deacetylase (CHR-3996, CHF-2845, 4SC-202, etc.) and HSP90 (IPI-504, AUY922, STA-9090, etc.), are under evaluation in ongoing clinical trials (79). Accumulating evidence suggests that CREB/ATF family proteins act as signaling molecules activated in response to stress (Table 1). In various cancers, CREB protects tumor cells from IR in others it promotes apoptosis. Different approaches (CREB mutants, CRE decoy oligonucleotides) and a number of small molecules (Ro 31-8220, NSC 12155, NSC 45576, naphthol AS-E phosphate) have been used to inhibit CREB function and transcriptional activity, as recently reported in a comprehensive review (80). Nevertheless, according to http://ClinicalTrials.gov website no clinical trials combining CREB inhibitors with RT are currently ongoing. The combination of CREB inhibition and IR can be certainly considered as a promising RT approach but a careful evaluation of appropriate conditions is needed before going from bench to bedside. Indeed, we think that further preclinical work is still necessary to identify and better characterize the molecular networks involved by this pleiotropic actor in exerting cell death or cell survival in response to IR treatment of cancer cells.

**Table 1:** **Involvement of cyclic AMP response element binding (CREB) pathway in different types of cancer cells after exposure to X-ray ionizing radiation (IR) treatment**.

Type of cancer	Pathway(s)	IR dose (Gy)	Effect	Reference
Hematological (erythro-leukemia)	AKT/CREB	1.5–6	Radio-resistance by increasing CREB phosphorylation	(5)
Hematological (Ramos B lymphoma cells)	ATF/CREB	3–5	CREB-related apoptosis	(6)
Gastrointestinal (colon cancer cells)	ATF2/CREB/CREM	6	Increase cytoplasmic phospho-CREB expression, suppress apoptosis	(64)
Gastrointestinal (colonic crypts)	ATF2/CREB/CREM	10	Increase cytoplasmic phospho-CREB expression	(67)
Lung (H1299 human lung cancer cells)	AKT/CREB	0–8	CREB-related apoptosis	(75)
Lung (non-small cell lung cancer cells)	EGFR-p38/ERK STAT3/CREB-1 epithelial/mesenchymal transition	2–6	Migration/invasion	(76)
Prostate (prostate cancer cell line LNCaP)	ATF-2/CREB	5–20	Neuroendocrine differentiation (NED)	(77)
			Radio-resistance by increasing CREB phosphorylation at 10 Gy	
Prostate (prostate cancer cell line LNCaP-HA-ACREB)	ATF-2/CREB	2–10	NED	(78)
			Radio-resistance by increasing CREB phosphorylation	

## Author Contributions

FDA: study and analysis of scientific literature and preparation of the manuscript and Table 1. LC: critical reading of the manuscript. MC: preparation of the reference list and Table 1. AA: analysis of the manuscript. RDP: supervision, correction, and improvement in the preparation of the manuscript and Table 1.

## Conflict of Interest Statement

This review has been done in the absence of any commercial or financial relationships that can be considered as a potential conflict of interest.
